# Epistaxis as a marker for severe acute respiratory syndrome coronavirus-2 status – a prospective study

**DOI:** 10.1017/S0022215120001863

**Published:** 2020-08-25

**Authors:** MH Hussain, M Mair, P Rea

**Affiliations:** Department of Otolaryngology, University Hospitals of Leicester NHS Trust, UK

**Keywords:** COVID-19, Coronavirus, Epistaxis

## Abstract

**Objective:**

To evaluate the prevalence of severe acute respiratory syndrome coronavirus-2 infection in patients presenting with epistaxis to a tertiary otolaryngology unit.

**Methods:**

A prospective study was conducted of 40 consecutive patients presenting with epistaxis referred to our tertiary otolaryngology unit. A group of 40 age-matched controls were also included. All patients underwent real-time reverse transcriptase polymerase chain reaction testing for severe acute respiratory syndrome coronavirus-2. Symptoms of fever, cough and anosmia were noted in the study group.

**Results:**

The mean age was 66.5 ± 22.4 years in the study group. There were 22 males (55 per cent) and 18 females (45 per cent). The mean age in the control group was 66.3 ± 22.4 years (*p* = 0.935). There were six positive cases for severe acute respiratory syndrome coronavirus-2 (15 per cent) in the epistaxis group and one case (2.5 per cent) in the control group. The difference was statistically significant (*p* = 0.05).

**Conclusion:**

Epistaxis may represent a presenting symptom of severe acute respiratory syndrome coronavirus-2 infection. This may serve as a useful additional criterion for screening patients.

## Introduction

Severe acute respiratory syndrome coronavirus-2 (SARS-CoV-2) is a pandemic coronavirus that causes the coronavirus disease 2019 (Covid-19) syndrome. Whilst in most cases only a mild illness ensues, severe disease can be complicated by acute respiratory distress syndrome, septic shock, cardiac injury and death. Initial studies reported the most common symptoms to be cough and fever. Other reported symptoms included shortness of breath and diarrhoea.^1^

A significant finding has been the emergence of anosmia as a key symptom for SARS-CoV-2 infection.^2^ A multicentre European study reported olfactory dysfunction in 85.6 per cent of patients with mild to moderate Covid-19.^3^ Within the respiratory tract, nasal epithelial cells demonstrate the highest expressions of the SARS-CoV-2 receptor, angiotensin-converting enzyme 2.^4^ Furthermore, previous strains of coronavirus have been found to propagate within the olfactory bulb and invade the central nervous system through olfactory epithelium.^5^ One proposed theory for the underlying pathophysiological process of anosmia in Covid-19 is viral-induced inflammation causing damage to cells in the nasal epithelium which are responsible for olfactory function.^6^

Given the established impact of SARS-CoV-2 on nasal mucosa, it is possible that such inflammation increases the risk of epistaxis. Inflammatory conditions affecting the nasal epithelium such as allergic rhinitis and chronic rhinosinusitis are clear risk factors for epistaxis, and the question arises as to whether SARS-CoV-2 has a similar effect.^7,8^

No prospective studies to date have investigated epistaxis as a presenting symptom for Covid-19. A retrospective study of 54 patients with anosmia and confirmed Covid-19 reported that 6 patients (11 per cent) had epistaxis.^9^ A further retrospective study of 20 Covid-19 patients requiring ENT consult found that 6 patients (30 per cent) presented with epistaxis.^10^ Current national UK data collected by the UK Trainee Research Collaborative Network, Integrate, revealed that 17.2 per cent of patients presenting with epistaxis tested positive for SARS-CoV-2.^11^ Our Trust covers a population of 706 155 patients. There have been 1338 confirmed cases (1.9 per cent) of Covid-19 in the region as of 7 June 2020.^12^

If epistaxis is a marker of SARS-CoV-2 infection, it is of critical importance that healthcare practitioners are aware of this and are provided with appropriate personal protective equipment. Furthermore, if a high prevalence of SARS-CoV-2 infection is identified in patients presenting with epistaxis, this may be a new marker of potential infection requiring self-isolation. This prospective study was designed to determine the prevalence of SARS-CoV-2 infection in patients presenting with epistaxis to a tertiary otolaryngology department.

## Materials and methods

This study was approved by the audit committee of the University Hospitals of Leicester NHS Trust. Patients were eligible if they were referred to our otolaryngology department with an episode of epistaxis. This was either patients presenting to the emergency department with epistaxis, or in-patients with epistaxis requiring an otolaryngology consult. Children were included in the study and there were no exclusion criteria based on the aetiology of epistaxis.

Each patient underwent a combined nasopharyngeal and throat swab for SARS-CoV-2 at the time of presentation. The swab, taken by a qualified nurse, was inserted into the oropharynx and rotated twice around the tonsil area. The same swab was then inserted 1–1.5 cm into one nostril and rotated for 3 seconds. Real-time reverse transcriptase polymerase chain reaction was used to confirm SARS-CoV-2 infection. The culture swab used at our institute is a Sigma Virocult®, a small vial with 1.0 ml medium and a standard Sigma swab. The management of epistaxis was unaffected by this study.

Demographic information was collected. At presentation, the medical history of each patient was checked to assess the use of anticoagulants, risk factors for epistaxis and history of recurrent epistaxis. Patients were also screened for SARS-CoV-2 symptoms. Recent symptoms of anosmia, fever and cough were recorded. The data for 40 consecutive patients were collected.

Data for a control group were also collected. For each patient presenting with epistaxis, the next patient to present to the emergency department within the same week and of the same age (± 1 year) with a non-infective condition was used as a control. The swab results for these patients were reviewed.

For both the study and control groups, any repeated swab results sent within two weeks from the original swab result were reviewed. Any subsequent SARS-CoV-2 testing occurred on the request of the caring team and was independent of this study.

Statistical analysis was performed using SPSS® software. The Mann–Whitney U test was used for asymmetrically distributed variables and Fisher's exact test was used to compare binary variables. A *p*-value of 0.05 or less was considered statistically significant.

## Results

Between 27 April 2020 and 28 May 2020, 40 patients with epistaxis were included in this prospective study. The data for 40 patients in the control group were also collected. Table 1 shows the demographic data for patients in both the study and control groups. In the study group, the mean age was 66.5 ± 22.4 years. There were 22 males (55 per cent) and 18 females (45 per cent). In the control group, the mean age was 66.3 ± 22.4 years. There were 17 males (42.5 per cent) and 23 females (57.5 per cent).

**Table 1.** Demographic details

Demographics	Study group*	Control group^†^	*P*-value
Age (mean ± SD; years)	66.5 ± 22.4	66.3 ± 22.4	0.935
Male (*n* (%))	22 (55)	17 (42.5)	0.301
Female (*n* (%))	18 (45)	23 (57.5)	0.301

*n = 40; ^†^*n* = 40; SD = standard deviation

The number of epistaxis patients on anticoagulant medication was 19 (47.5 per cent). In 14 patients (35 per cent), there was a history of recurrent epistaxis, whilst for the remaining 26 patients (65 per cent), the epistaxis was a one-off episode. There were pre-existing risk factors for epistaxis in four patients (10 per cent). These included septal perforation, allergic rhinitis and granulomatosis with polyangiitis (Table 2).

**Table 2.** Epistaxis risk factors and Covid-19 symptoms

Parameter	Patients (total *n*)	SARS-CoV-2 positive (*n* (%))	SARS-CoV-2 negative (*n* (%))	*P*-value
Risk factors for epistaxis				
– Using anticoagulants	19	3 (15.8)	16 (84.2)	0.619
– History of recurrent epistaxis	14	2 (14.2)	12 (85.7)	0.654
– Other risk factors for epistaxis	4	1 (25.0)	3 (75)	0.493
Clinical symptoms of Covid-19				
– Persistent cough	5	0 (0)	5 (100)	0.423
– Persistent fever	3	1 (33.3)	2 (66.6)	0.394
– Anosmia	2	1 (50)	1 (50)	0.281

Covid-19 = coronavirus disease 2019; SARS-CoV-2 = severe acute respiratory syndrome coronavirus-2

In terms of symptoms of SARS-CoV-2 infection, five epistaxis patients (12.5 per cent) had a history of persistent cough, none of whom tested positive for SARS-CoV-2. Of the three patients (7.5 per cent) who reported persistent fevers, one patient tested positive. Of the two patients (5 per cent) who reported a history of anosmia, one tested positive (Table 2).

In the epistaxis group, four patients (10 per cent) tested positive for SARS-CoV-2 at initial presentation. Two patients, who initially tested negative, subsequently tested positive on reverse transcriptase polymerase chain reaction, giving a total of six positive cases. Of the six epistaxis patients who tested positive for SARS-CoV-2, none reported a history of fever; one patient reported a recent cough and one patient reported anosmia. In the control group, no patients tested positive on initial presentation to the emergency department. One patient (2.5 per cent) subsequently tested positive for SARS-CoV-2 following admission. The difference between the study group and the control group was statistically significant (*p* = 0.05) (Table 3).

**Table 3.** SARS-CoV-2 status in study and control groups

Group	SARS-CoV-2 positive (*n* (%))	SARS-CoV-2 negative (*n* (%))	*P*-value
Epistaxis*	6 (15)	34 (85)	0.05
Control^†^	1 (2.5)	39 (97.5)	

*n = 40; ^†^*n* = 40. SARS-CoV-2 = severe acute respiratory syndrome coronavirus-2

## Discussion

The theoretical risk of epistaxis in patients with Covid-19 emerged with evidence relating to anosmia in patients with coronavirus. Studies on previous strains of coronavirus have demonstrated that healthy individuals with no history of olfactory dysfunction can develop anosmia after exposure to the virus.^13^ A recent study demonstrated that, within the respiratory tract, the nasal respiratory epithelium has the highest expression of SARS-CoV-2 entry genes.^4^ A study in 2008 on transgenic mice for the SARS-CoV receptor, angiotensin-converting enzyme 2, found that the entry point for brain infections was the olfactory bulb. Furthermore, the virus antigen was most abundant at the olfactory bulb.^14^

Collectively, these studies demonstrate that the nasal mucosal lining is affected by coronavirus. The exact underlying pathophysiology is not clearly understood. A recent study on olfactory tissue samples from humans and mice suggested that the SARS-CoV-2 receptors are expressed specifically in non-neuronal cells of the olfactory epithelium.^6^ The authors suggested that an inflammatory or destructive process affecting support cells mediated the altered function of olfactory sensory neurons, leading to anosmia.^6^ Inflammation of the nasal epithelium mediated by SARS-CoV-2 may increase the risk of epistaxis, as is the case with allergic rhinitis or chronic rhinosinusitis.^7,8^ Identifying whether SARS-CoV-2 may present with epistaxis is important because it may add an important screening tool, especially as issues remain around reverse transcriptase polymerase chain reaction sensitivity and availability.

This is the first study to prospectively evaluate the prevalence of SARS-CoV-2 infection amongst patients with epistaxis to determine whether there is a significant association. The incidence of SARS-CoV-2 infection amongst epistaxis patients was 15 per cent, compared to 2.5 per cent in the control group. This demonstrates a significant difference between the groups (*p* = 0.05). The rate of positive cases in our study is comparable to the interim data released by the UK Trainee Research Collaborative Network, Integrate. In their audit of 567 epistaxis patients, swabs for SARS-CoV-2 were taken in 93 patients, of which 16 tested positive (17.2 per cent).

In our methodology, we only included cases of SARS-CoV-2 confirmed on reverse transcriptase polymerase chain reaction testing. One patient tested negative but presented with symptoms highly suspicious of Covid-19. He presented with recent anosmia and upper respiratory tract infection symptoms, as well as epistaxis. He denied any previous history of epistaxis, was not on any anticoagulant drugs and had no other risk factors for epistaxis. This patient did not have any further reverse transcriptase polymerase chain reaction tests as his epistaxis was controlled and he was discharged home on the day of presentation. If considered positive, the number of positive cases would have been 7 out of 40 (*p* = 0.028).

- There is established evidence that severe acute respiratory syndrome coronavirus-2 (SARS-CoV-2) impacts nasal epithelium, possibly increasing the epistaxis risk
- A statistically significant difference was found in SARS-CoV-2 prevalence in epistaxis patients compared to a control group
- Epistaxis may be a potential marker for infection in SARS-CoV-2
- Caution and adequate personal protective equipment is needed when dealing with epistaxis patients given the potentially increased risk of SARS-CoV-2 infection

This study highlights issues surrounding the sensitivity of initial reverse transcriptase polymerase chain reaction testing. Of the six positive cases in our study, two patients (33.3 per cent) initially tested negative on admission but then subsequently tested positive. Among our small sample of six positive cases, the sensitivity of initial reverse transcriptase polymerase chain reaction testing was 66.6 per cent, if a positive result on repeat testing is considered the ‘gold standard’. This raises similar concerns to those of previous studies on reverse transcriptase polymerase chain reaction sensitivity.

In one study of 205 patients, the reported sensitivity of reverse transcriptase polymerase chain reaction for nasal swabs was 63 per cent and only 32 per cent for throat swabs.^15^ A systematic review reported the false negative rates of reverse transcriptase polymerase chain reaction testing to be 2–29 per cent (sensitivity of 71–98 per cent), based on negative reverse transcriptase polymerase chain reaction findings that are positive on repeated testing.^16^ Even these sensitivity rates are likely to be inflated given the inevitable incorporation bias involved in determining the sensitivity of a test (reverse transcriptase polymerase chain reaction) whilst using the same test (repeated reverse transcriptase polymerase chain reaction testing) as the gold standard.^17^

## Conclusion

Epistaxis may represent a presenting symptom of SARS-CoV-2 infection. Further clinical studies are needed to demonstrate this association. Such an association needs to be communicated to the medical community, as epistaxis may provide an additional important screening tool.
